# In vivo and in vitro effects of QHF combined with chemotherapy on hepatocellular carcinoma^☆^

**DOI:** 10.1016/S1674-8301(10)60025-5

**Published:** 2010-03

**Authors:** Chen Tao, Li Dan, Fuya ling, Gongzi Peng

**Affiliations:** Medical College of Three Gorges University, Yichang 443002, China

**Keywords:** Chinese medicine ingredients, QHF, hepatocellular carcinoma, cisplatin, apoptosis

## Abstract

**Objective:**

To investigate the synergistic anti-tumor effect of QHF (a Chinese medicine formula with anti-tumor active ingredients, including 800 mg/kg Cinobufotalin, 14 mg/kg Ginsenoside Rg3, 5.5 mg/kg Notoginseng and 100 mg/kg Lentinan) when combined with the chemotherapy drug cisplatin (DDP).

**Methods:**

Hepatocellular carcinoma H_22_ cells were implanted into mice and after the transplants were successfully established the animals were divided into four groups, namely a normal saline(NS) control group, QHF group, DDP group and QHF+DDP group. The tumor growth was monitored and the survival time determined. In vitro studies employing H_22_ cells used the first three groups, and determined the effects of QHF and DDP on tumor cell cycle distribution, apoptosis and morphologic changes *in vitro*.

**Results:**

QHF significantly inhibited the growth of tumors and prolonged the survival time of mice with hepatocellular carcinomas. QHF combined with DDP could attenuate DDP-induced leucopenia, spleen and thymus atrophy and other indicators of toxicity. The inhibition rate of tumor growth reached 82.54% with QHF+DDP, and QHF prolonged the life span of DDP-treated mice by 66.83%. In the *in vitro* experiments tumor cells showed morphological changes characteristic of apoptosis by both light and transmission electron microscopy in the QHF group, and the apoptosis rate was 33.85%. Moreover, the proportion of cells in the G_0_/G_1_ phase was increased and those in the S-phase decreased.

**Conclusion:**

QHF combined with DDP could significantly inhibit tumor growth, induce the apoptosis of tumor cells and effectively attenuate DDP toxicity.

## INTRODUCTION

Hepatocellular carcinoma (HCC) is one of the most prevalent tumor types and the third most common cause of cancer-related death worldwide. One 5-year survival rate report of patients with HCC was less than 5%. Moreover, epidemiological surveys show that HCC morbidity and mortality ranked third of all malignant tumors and over 50% of HCC worldwide occur in China. Furthermore, the mortality rate is different in the different geographical regions[1]. At present, the international treatment programs for HCC are divided into two categories: one is curative, and includes surgical resection and liver transplantation. Resection is currently indicated for patients with a solitary HCC and well-preserved liver function, who have neither clinically significant portal hypertension nor abnormal bilirubin. Liver transplantation benefits patients who have decompensated cirrhosis and one tumor smaller than 5 cm or up to three nodules smaller than 3 cm. However, donor shortage greatly limits its applicability. The other treatment program is called thermal ablation, and includes such interventional treatment as, percutaneous liver thermal ablation therapy[2], cryotherapy, ethanol injection[3], radiofrequency ablation, microwave ablation, and laser ablation[4]–[6]. To a certain degree, these palliative therapies not only partially improve the quality of life and prognosis of patients with HCC, but they also give them the opportunity for a subsequent surgical resection. In addition, treatment choices for unresectable HCC also include Raf kinase inhibitor protein[7], tyrosine kinase receptor inhibitors[8]–[10], immunotherapy[11],[12], and other methods and therapies[13]–[17]. As is well known, for patients with HCC, hepatic resection or transplantation are considered the standard of care, with 5-year survival rates ranging from 30% to 70%. However, unfortunately, less than 15% of patients are suitable for surgery due to multiple tumors, a tumor > 5 cm, inadequate liver function reserve, and proximity to and/or involvement of vascular or biliary structures. As a result the overall 5-year survival for HCC patients is less than 10%[18].

Chemotherapy has been less effective in its clinical application because of the traditional toxic side effects of the chemotherapeutic agents and the poor sensitivity of the HCC. Moreover, the application and promotion of interventional therapy and other new therapies for new targets are difficult in smaller hospitals, especially at the grassroots level hospital, because of their technical mastery, efficacy and other factors[19]–[22]. Thus, in recent years, many clinicians have often emphasized the use of traditional Chinese medicines for their synergistic effects and few side effects.

The compound recipe is still the mainstream of Chinese medicine prevention and treatment of HCC. Its overall and regulatory feature of multi-component, multi-link and multi-targets is of great importance for prevention and treatment of HCC which is characterized by multi-gene regulation, complex pathogenesis and multivariate pathogenetic conditions. However, in-depth studies on the mechanism of Chinese medicine compounds are greatly limited due to the complicated ingredients of the Chinese herbal compound and the unknown basis of the effective materials, making improvements in efficacy difficult. Therefore, in order to increase the role and status of Chinese medicine in HCC prevention and treatment, great importance should be placed on studying the mechanism of action of the active ingredients. In recent years, a new compatibility mode called the active ingredient of Chinese medicine compound, has become a hot research field that includes determining components and the pharmacodynamic relationships among them[23],[24]. However, there have been no reports on the application of traditional Chinese medicine compound components in the prevention and treatment of malignant carcinomas, especially on HCC. Under the theory of traditional Chinese medicine, Qingrejiedu (Q: clear away heat and toxin), Huoxuehuayu (H: promote blood flow to remove stasis) and Fuzhengguben (F: strengthen healthy qi and root) we have developed and used a formula called QHF as a major therapeutic method for HCC in the clinics. Since 2003, we have been screening Chinese medicine anti-tumor active ingredients, and found that the optimal QHF dose ratio for anti-HCC treatment was: 800 mg/kg Cinobufotalin, 14 mg/kg Ginsenoside Rg3, 5.5 mg/kg Notoginseng and 100 mg/kg Lentinan[25]–[27]. In previous studies, it was confirmed that for anti-HCC treatment this formula was more efficient than any of the single drugs that constitutes the formula, and that is has few side effects[25]. In the present study the aim was to further investigate the anti-HCC efficacy of QHF when combined with the chemotherapy drug DDP, and determine potential mechanisms of action.

## MATERIALS AND METHODS

### Drugs and reagents

Cinobufotalin was purchased from the Anhui Jinchan Biochemical Ltd., Lot No: 041103. Ginsenoside Rg3 was purchased from the Jilin Yatai Pharmaceutical Co, Ltd., Lot No: 050302. Notoginseng was purchased from the Yunnan Weihe Pharmaceutical Co, Ltd., Lot No: 050124. Lentinan was purchased from the Zhejiang Jinhua Essen Pharmaceutical Co, Ltd., Lot No: 050202. DDP was purchased from the Jinzhou Jiutai Pharmaceutical Co, Ltd., Lot No: 060302. The above drugs were all prepared to the required concentration with normal saline. Annexin V/FITC double staining kit was purchased from Hangzhou MultiSciences Biotech Co, Ltd..

### Animals and methods

#### Culture of H_22_ hepatoma cell

Under aseptic conditions, H_22_ hepatoma ascites tumor-bearing mice were sacrificed by cervical dislocation 7 days after intra peritoneal inoculation with H_22_ tumor cells (purchased from Shanghai Institute of Materia Medica, Chinese Academy of Sciences, and preserved by the Immune Research Center of our university). The thick and white ascites tumor cells were collected aseptically and stained by Trypan Blue to microscopically determine their viability, which was more than 95%. H_22_ cells were diluted with NS and the cell concentration was adjusted to 5×10^5^/ml.

#### Animal treatment and experimental design

Animal experiments were performed in accordance with the Guide for the Care and Use of Laboratory Animals of Hubei Province and approved by the local Ethics Committee.

Balb/c mice were provided by the Laboratory Animal Center of Huazhong University of Science and Technology, Tongji Medical School (certificate number SCXK (E) 2003-2005). Male Balb/c mice (18-20 g) were reared in a temperature and humidity-controlled environment under a 12-h light/12-h dark cycle, and given food and water ad libitum.

Under aseptic conditions, the mice were immobilized and the right axillary skin disinfected with alcohol. Then the mice were inoculated with 0.2 ml medium containing 2×10^4^ tumor cells in the right axilla to establish the solid tumor model, and the same number of the cells were inoculated into the peritoneum to establish the ascites tumor model. The experimental mice were randomly divided into four groups as follows: NS (saline control) group, DDP group (3 mg/kg), QHF group (Cinobufotalin 800 mg/kg, Ginsenoside Rg3 14 mg/kg, Notoginseng 5.5 mg/kg and Lentinan 100 mg/kg, a formula created by Prof Chen Tao), and QHF+DDP group (using the same doses of QHF and DDP). All drugs were administered intraperitoneally once a day for 10 days. The control group was given the same volume of vehicle. The experiment was carried out on the 10^th^ day of drug therapy.

### Inhibition rate of tumor growth

Twenty-four hours after the last drug administration the mice were sacrificed (solid tumor model) by cervical dislocation. Then the tumors were removed and weighed. Inhibition rate was calculated by the following formula: Inhibition rate (%) = {[tumor weight of NS group (g) - tumor weight of drug group (g)] / tumor weight of NS group (g)} ×100%.

### The survival rate of mice

After continuous administration of therapy for 10 days we observed the condition of mice with ascitic tumors and recorded their survival. The survival rate was calculated according to the following formula: The survival rate (%) = [average survival in days of drug group - average survival in days of the NS group)/average survival in days of the NS group] ×100%.

### Changes in body weight and in the immune system

#### Weight of mice

Tumor model mice were weighed before and twenty-four hours after the last administration of drugs, and the changes of body weight during treatment were computed.

#### Counting of WBC

Before killing the mice with the ascites tumors we took blood from the tail vein to perform a conventional white blood cell count.

#### Thymus and spleen index

Twenty-four hours after the last drug administration, the ascites tumor bearing mice were sacrificed by cervical dislocation. We removed the thymus and spleen and weighed them on an electronic balance. The thymus or spleen index was calculated by the following formula: thymus (spleen) index = weight of thymus (spleen) (mg)/weight of mouse (g).

### Morphological examination of tumor cells

#### Light microscopy

The abdominal skin of each mouse was disinfected with alcohol and the ascites fluid removed, then centrifuged and the sediment collected. Each sediment was then smeared, stained and observed by light microscopy. The morphological examination of all samples was performed by a single investigator who was blind to the experimental groups.

#### Ultrastructure using transmission electron microscopy

Epon embedded, ultrathin sections were stained with uranyl acetate-lead citrate and observed by transmission electron microscope.

### Tumor cell apoptosis and cell cycle detection

#### Detection of apoptosis

Apoptosis of mouse hepatocellular carcinoma H_22_ cells was detected by flow cytometry. Mouse H_22_ hepatocellular carcinoma cells were seeded at 1.5×10^5^ per well in 6-well plates. After 48 h, cells were treated with the appropriate drugs for 24-72 h in complete medium. Cells were harvested with trypsin, washed twice with cold PBS before adding 5 µl Annexin-V and 10 µl propidium iodide (PI) while avoiding light. Cells were incubated at room temperature for 15 min, and buffer added to 200 µl prior to detection by flow cytometry.

#### Analysis of the cell cycle distribution

Mouse H_22_ hepatocellular carcinoma cells were seeded at a density of 1×10^5^ in 60-mm culture dishes. After 24 h, cells were treated with the appropriate drugs for 24-72 h in complete medium. At the indicated time, cells were harvested by trypsinization, centrifuged at 2,000 rpm for 5 min, washed in cold PBS, fixed overnight in 75% cold ethanol, digested with 20 µg/ml RNase A and stained with 50 µg/ml PI. The cells were then subjected to flow cytometric analysis on the FACSCalibur flow cytometer (Beckman Coulter EpicsXL-4 System II, USA).

### Statistical analysis

All measurement data are expressed as mean±SD. Data were analyzed using the SPSS 10.0 software package. Differences among groups were analyzed by one-way analysis of variance (ANOVA) and post hoc Tukey's tests were used for multiple comparisons. Chi-square test was used to test the differences for enumerative data among groups, and Qatar segmentation was used in the comparison between two factors. The P-value reported was two-sided and values of *P* < 0.05 were considered statistically significant.

## RESULTS

### General condition of mice and tumor growth inhibition

Within five days of inoculation, there were no significant changes in the appearance of the mice. Behaviorally, for about one hour after the daily gavage, the mice crowded together, and then gradually returned to their normal distribution in the cage. One week later, the subcutaneous mass on the right axilla was palpable and the tumors in the solid tumor model group grew rapidly thereafter, and there were obvious signs of local swelling and pressure. The tumors of mice in the drug-treated group grew slowly with an elliptical shape, and while there was significant piloerection and reduced activity, these were clearly better than in the untreated tumor model group. Mice in the DDP group showed evidence of drug toxicity, such as anorexia, diarrhea, emaciation and superficial skin peeling. On the other hand, mice in the QHF+DDP group did not show obvious signs of drug toxicity, and they had no significant weight loss or decreased activity and their feeding behavior appeared normal.

There was a significant (*P* < 0.01) inhibition of tumor growth in the QHF, QHF+DDP and DDP groups when compared to the control NS group. The inhibition rates were 55.53%, 82.54%, 74.28%, respectively. The inhibition rate of the QHF+DDP group was significantly higher than that of the other groups (*P* < 0.01, ***Table 1***).

**Table 1:** Effects of QHF and DDP, separately and together, on tumor weight and inhibition rate in the tumor-bearing mice

Group (*n* = 10)	Weight of tumor(g)	IR(%)
NS	1.290 ± 0.118^##^	
DDP	0.655 ± 0.056**	74.28
QHF	0.857 ± 0.113**^##^	55.53
QHF+DDP	0.538 ± 0.061**	82.54

**P < 0.01 vs NS group. ^##^*P* < 0.01 *vs* DDP group.

### General condition and survival of mice

Six days after tumor cell inoculation, abdominal ascites and piloerection became obvious, and there was a significant reduction of the activity of mice in the NS group, and these effects steadily increased with time. Eight days after inoculation, the formation of abdominal ascites was also observed in mice of the DDP and QHF group animals, but the ascites accumulation increased slowly. The mice in the DDP group became emaciated and lacked energy. The formation of abdominal ascites was seen in mice in the QHF+DDP group just after stopping drug administration, and this increased slowly by 15 days after tumor cell inoculation. The mice were not thin and their activity and feeding behavior were good. But 15 days later, their activity was less and feeding decreased. Piloerection was evident and there was an increase in the rate of ascites accumulation.

The survival times of mice were significantly extended in the QHF and QHF+DDP groups, 38.46%, 87.02% respectively. There was a significant difference compared with the NS group (*P* < 0.01). The life span of mice in the QHF group was significantly higher than that of the DDP group (*P* < 0.01) (***Table 2***).

**Table 2:** Effects of QHF and DDP, separately and together, on survival time and life span change in the tumor-bearing mice

Group (*n* = 10)	Survival time (days)	Life span (%)
NS	20.80 ± 2.440^##^	
DDP	25.00 ± 1.564**	20.19
QHF	28.80 ± 2.821**^##^	38.46
QHF+DDP	38.90 ± 1.969**^##^	87.02

**P < 0.01 vs NS group. ^##^*P* < 0.01 *vs* DDP group.

### Toxicity of drugs in tumor-bearing mice

#### Body weight of the tumor-bearing mice

The weights of the tumor-bearing mice were significantly increased in the QHF group (*P* < 0.01), while those of the DDP group animals decreased (*P* < 0.01). The increase in mean weight of the QHF+DDP group was obviously higher than that of DDP group (*P* < 0.01)(***Table 3***). These data showed QHF had a significant role in increasing the weight of tumor-bearing mice receiving chemotherapy.

**Table 3:** Effects of QHF and DDP, separately and together, on the weight of the tumor-bearing mice

Group (*n* = 10)	Increase in body weight(g)
NS	1.727 ± 0.393^##^
DDP	0.842 ± 0.213**
QHF	2.711 ± 0.348**^##^
QHF+DDP	2.233 ± 0.465*^##^

*P < 0.05, **P < 0.01 vs NS group. ^##^*P* < 0.01 *vs* DDP group.

### Changes of WBC in peripheral blood and spleen and thymus weights in tumor-bearing mice

Compared with the NS group, the number of WBC of the DDP and QHF+DDP animals were significantly lower (*P* < 0.01). There was no difference between the QHF and NS groups, and the QHF+DDP group mean value was higher than that of the DDP group (*P* < 0.05) (***Table 4***).

**Table 4:** Effects of QHF and DDP, separately and together, on WBC in peripheral blood and thymus and spleen indexes of the tumor-bearing mice

Group (*n* = 10)	Number of WBC (×10^9^/L)	Index of thymus (mg/g)	Index of spleen (mg/g)
NS	10.6580 ± 1.815^##^	0.8100 ± 0.200^##^	7.9600 ± 1.826^##^
DDP	5.7700 ± 0.936**	0.4780 ± 0.080**	5.5980 ± 1.059*
QHF	9.0750 ± 0.948^##^	1.0300 ± 0.156*^##^	10.5500 ± 1.833*^##^
QHF+DDP	7.8300 ± 0.976**^#^	0.8810 ± 0.130^##^	9.9100 ± 1.986^#^

*P < 0.05, **P < 0.01 vs NS group. ^#^*P* < 0.05, ^##^*P* < 0.01 *vs* DDP group.

The weights of the thymus and spleen in tumor-bearing mice were only increased in the QHF group (*P* < 0.05), while the weights of the thymus (*P* < 0.01) and spleen (*P* < 0.05) were significantly decreased in the DDP group. When comparing the DDP and QHF+DDP groups, we found that the thymus and spleen weights of mice receiving chemotherapy could be improved by QHF(*P* < 0.05 and *P* < 0.01 respectively) (***Table 4***).

### Tumor cells morphological observation

**Fig. 1:**
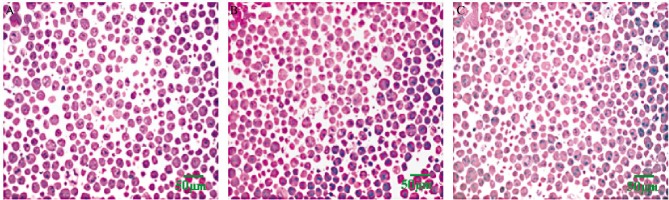
The representative photomicrographs under light microscope of tumor cells in the tumor-bearing mice (HE,×400). A: NS group. B: DDP group. C: GHF group.

Under light microscopy, we saw that the tumor cells of the NS group were round or oval in shape and the nuclei were large and round. Furthermore, the morphologic changes seen were independent of cell size, and included nuclear chromatin condensation, a large nucleus-cytoplasm ratio, more nuclei, and more pathological karyokinesis. The tumor cells of the QHF group were smaller in size and karyokinesis was relatively less. Moreover, indications of steatosis and surface sprouting were observed. Cells from the DDP group showed extensive edematous degeneration, hyperchromatic nuclei, nuclear fragmentation and a large number of cell exhibited necrosis (***Fig. 1***).

**Fig. 2:**
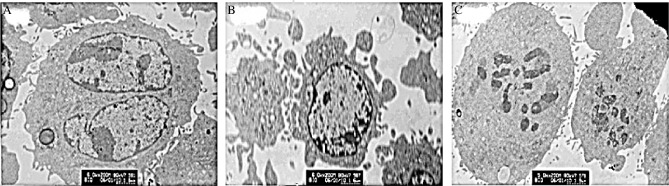
The representative photomicrographs (×6000) of TEM images of tumor cells from the tumor-bearing mice. A: NS group. B: DDP group. C: GHF group.

Using electron microscopy, we found that tumor cells from the NS group animals were large and round. Chromatin was evenly distributed and was of the same density. Also, integral karyotheca, rich microvilli on the plasma membrane and normal organelles were observed in tumor cells from the NS group. Surface microvilli of tumor cells from the QHF group animals disappeared and the chromatin was condensed, coagulated and scattered within the membrane. Moreover, partial nuclear fragmentation, cytoplasm concentration, membrane shrinkage and evagination with budding, and mitochondrial swelling and deformation were observed in tumor cells from the QHF group. Tumor cell chromatin of the DDP group animals was more condensed, and decomposition and nuclear fragmentation were observed. The cytoplasm of some cells dissolved freely and cell necrosis also occurred in the DDP group tumor cells (***Fig. 2***).

### Rate of apoptosis of H_22_ cells

**Fig. 3:**
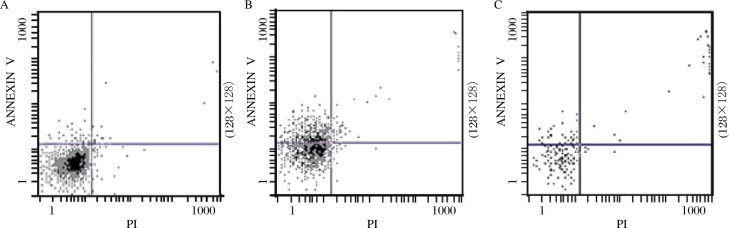
The representative photographs of the H_22_ cell apoptosis rate in the tumor-bearing mice. A: NS group; B: DDP group; C: QHF group. In each plot, the lower left(LL) quadrant represents viable cells, the upper left(UL) quadrant denotes early apoptotic cells, the lower right(LR) quadrant indicates necrotic cells, and the upper right(UR) quadrant represents necrotic or late apoptotic cells. The upper left(UL) quadrant were regarded as apoptotic cells and were used to calculate apoptosis rate.

Compared with the control group, apoptosis of tumor cells was significantly increased in the QHF and DDP groups and the rates of apoptosis were 33.85% and 39.04%, respectively, compared to the NS rate of 5.85%(*P* < 0.01). Although the mean QHF group apoptosis rate was lower than that of the DDP, the difference was not significant (*P* > 0.05) (***Table 5******,*** ***Fig. 3***).

**Table 5:** Effects of QHF and DDP on H_22_ cell apoptosis rate of the tumor-bearing mice

Group (*n* = 10)	Rate of apoptosis (%)
NS	5.85 ± 2.823
DDP	39.04 ± 8.502**
QHF	33.85 ± 5.106**

**P < 0.01 *vs* NS group.

### Cell cycle of the H_22_ cell

Tumor cells from the NS group animals proliferated rapidly, and the cells were mainly found in the S/G_2_-M phase. The proportion of G_0_/G_1_ phase cells of the QHF group tumor cells was increased compared with NS group cells (*P* < 0.01), showing that QHF could lead to the accumulation of cells in G_0_-G_1_ phase and prevent the transition to the S phase. Moreover, DDP could also increase cells in G_0_-G_1_ phase, compared with the NS group (*P* < 0.01) (***Table 6***).

**Table 6:** Effects of QHF and DDP on the H_22_ cell cycle of the tumor-bearing mice

Group (*n* = 10)	G_0_/G_1_(%)	G_2_/M(%)	S(%)
NS	29.29 ± 0.831	17.30 ± 0.842	52.90 ± 1.424
DDP	41.00 ± 1.076**	13.05 ± 0.727	46.20 ± 1.200
QHF	37.16 ± 1.198**	18.10 ± 0.612	44.58 ± 0.814

**P < 0.01 *vs* NS group.

## DISCUSSION

The development of HCC is a highly complex process involving numerous pathological and molecular mechanisms at the various stages of growth and metastasis, which makes a single effect target drug a poor choice. However, Chinese medicine has the feature of an integrated anti-HCC effect using a multicomponent, multisystem and multitarget approach. QHF is a new Chinese compound composed of four anti-tumor active ingredients according to a certain law of compatibility.

In vivo study we found that QHF could improve the general condition of tumor-bearing mice, alleviate signs and symptoms to a certain degree, significantly inhibit the growth of transplanted H_22_ tumors, and extend the survival of mice with tumor. Meanwhile, we also found that QHF combined with the chemotherapy drug DDP could attenuate the indicators of DDP-induced toxicity such as anorexia, diarrhea, weight loss, leucopenia and the shrinkage of the thymus and spleen. More importantly, QHF could increase the anti-tumor effect of DDP. Experimental results showed that it not only increased the DDP-induced inhibition rate by 8.26%, reaching 82.54%, but also extended the life span by 66.83%, reaching a life span of for QHF+DDP of 87.02%. Body weight is one of the comprehensive indicators for measuring the toxicity of a drug. The QHF group of tumor-bearing mice exhibited an increase in body weight compared to the NS tumor-bearing animals, while the body weight of the DDP group of animals was significantly lower, even showing a weight loss. Some animals had died before the DDP withdrawal, indicating that chemotherapy had failed, perhaps due to the side effects, but this did not happen in the QHF group.

Morphological observation is one of the most reliable ways to observe apoptosis. In this study, we collected ascites tumor cells from mice in each group. Under the light microscope, certain characteristics of apoptosis were observed in cells from the QHF and DDP groups. The tumor cells from the QHF group were smaller in size and mitosis was relatively less. In addition, some steatosis and surface sprouting were also observed. However, we found that extensive edematous cell degeneration, hyperchromatic nuclei, nuclear fragmentation and a large number of necrotic cells occurred in DDP group.

Under the transmission electron microscope, tumor cells from the NS group were large and round. Moreover, chromatin was evenly distributed and had the same density. Also, integral karyotheca, rich microvilli on the membrane and normal-appearing intracellular organelles were observed in tumor cells from the NS group. In tumor cells from the QHF group the surface microvilli disappeared and chromatin was condensed, coagulated and scattered around the membrane. Moreover, partial nuclear fragmentation, cytoplasm concentration, membrane shrinkage and evagination with budding, and intracytoplasmic mitochondrial swelling and deformation were also observed in tumor cells from the QHF group. Chromatin in the DDP group tumor cells appeared more dense and showed accumulation, decomposition, and nuclear fragmentation. In addition, cytoplasm of some cells dissolved freely and cell necrosis occurred.

We further detected the apoptosis ratio of tumor cells by flow cytometry by the Annexin/V staining method[28],[29], and found that tumor cell apoptosis could be significantly induced in the QHF and DDP groups with apoptosis rates of 33.85% and 39.04%, respectively. These were significantly different from the NS group (*P* < 0.01). Combined with morphological observations showing that QHF could indeed induce apoptosis of H_22_ hepatoma cells, we also detected changes in the cell cycle showing that the proportion of cells in the G_0_/G_1_ phase increased and the proportion in the S phase decreased in the QHF group. QHF could prevent cells entering the S phase and decrease the proliferative capacity of tumor cells, which would have a direct inhibitory effect on tumor cell growth. This suggests that one of the possible mechanisms of the anti-tumor effect of QHF is by inducing cell apoptosis by cell cycle arrest.

## CONCLUSION

This study indicated that in mice implanted with hepatocellular carcinoma cells, QHF could significantly inhibit the growth of tumors, and prolong the survival time and induce apoptosis of tumor cells. Moreover, when combined with DDP, QHF had a synergistic anti-tumor effect with this chemotherapeutic agent while attenuating DDP-induced toxicity. The combination of QHF and DDP might generate comprehensive effect and induce the apoptosis of hepatocellular carcinoma by using multicomponent, multisystem Chinese medicine with multiple targets. The study of the underlying mechanisms of the effect of QHF is being further investigated.
